# Lymphomatoid Papulosis Type A: A Case Report of the “Wait-and-See Strategy” in a 27-Year-Old Male Patient with Extensive Disease

**DOI:** 10.1155/2019/1765210

**Published:** 2019-07-16

**Authors:** Eirini Kavvalou, Maria Stefanidou, Sabine Elke Krueger-Krasagakis, George Evangelou, Dimitra Koumaki, Leonidas Marinos, Maria Tzardi, Konstantinos Krasagakis

**Affiliations:** ^1^Department of Dermatology, University General Hospital of Heraklion, 71500 Heraklion, Greece; ^2^Department of Haematopathology, Evaggelismos General Hospital of Athens, Ipsilantou 45-47, 10676, Athens, Greece; ^3^Department of Pathology, University Hospital of Heraklion, 71500 Heraklion, Greece

## Abstract

This is the case of a 27-year-old male patient with a newly diagnosed extensive lymphomatoid papulosis type A involving cosmetically sensitive areas (e.g. face), who refused to be treated with a low dose of methotrexate, as recommended by the published literature. The natural course of the disease was proved to be strikingly satisfying though, with a complete regression of all skin lesions at the 4-week follow-up, including an ulcerated nodule 3 x 3 cm in dimension, which was expected to heal at least in months. We report this case to reconsider weighting the risks and the short-term benefits of methotrexate treatment, even in the case of an extensive disease.

## 1. Introduction

Lymphomatoid papulosis is a rare, recurrent, self-healing papulonodular skin eruption and part of the group of cutaneous CD30^+^ lymphoproliferative disorders (LPDs). It is associated with a lifelong increased incidence of malignant lymphoma [1]. In case of an extended disease, treatment with methotrexate in a low dose is recommended in order to hasten the regression of the lesions [2]. In this case report a 27-year-old patient with extensive disease refused to take methotrexate because of its potential harmful side effects. The result in the 4-week follow-up was strikingly satisfying with the majority of papulonodular lesions having regressed, living hyperpigmented scarred lesions. Of interest has been the spontaneous regression of a nodular, ulcerated lesion with a diameter of 3 × 3 cm. Our case highlights the significance of weighting the potential harmful effects of methotrexate treatment against the possibility of a spontaneous regression even in terms of an extensive lymphomatoid papulosis.

## 2. Case Presentation

**Figure 1:**
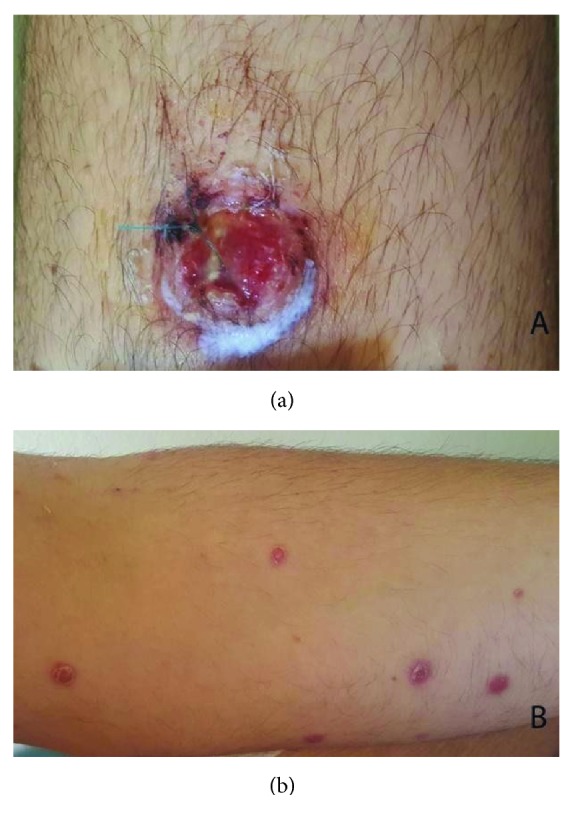
A large ulcerated 3 × 3 cm tumor on the extensor surface of the left thigh at presentation (a); a widespread erythematous papulonodular eruption involving the face, the trunk and the extremities with occasional scaling at the periphery of the lesions (b).

**Figure 2:**
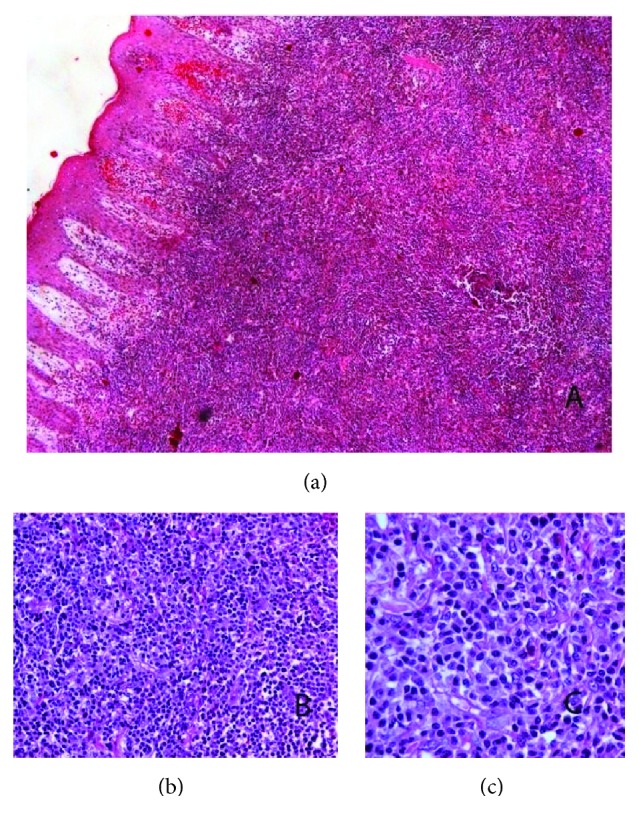
Histology revealed a diffuse lymphocytic infiltration in the reticular dermis by small, medium, and large sized lymphocytes, some of which with anaplastic nuclei, accompanied by a variable number of neutrophils and histiocytes (hematoxylin-eosin stain x 20 (a), x 40 (b), x 60 (c)).

**Figure 3:**
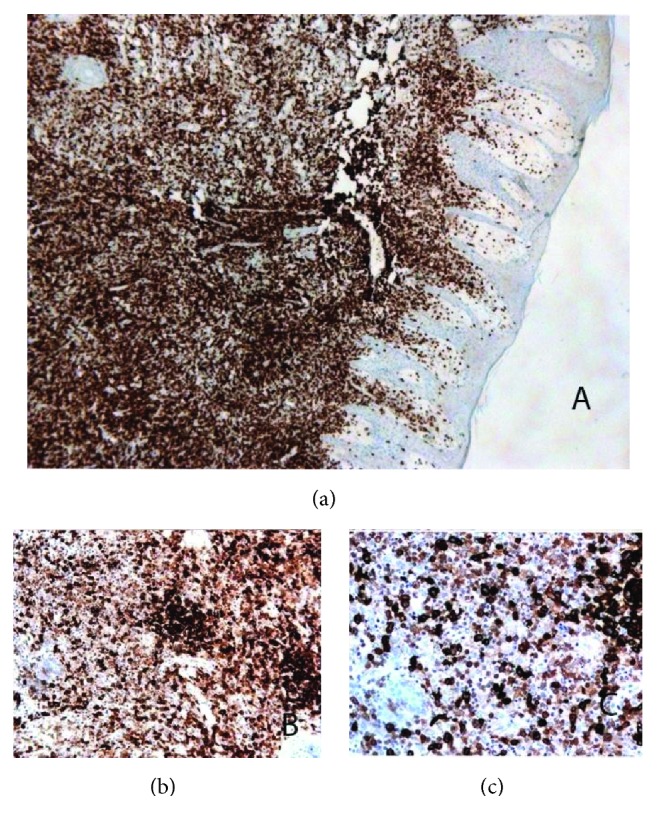
Immunophenotypically the lymphocytes were CD3+ (x20) (a) and >75% CD30+ (x40) (b) and (x60) (c) (Immunohistochemistry with Envision Detection System Peroxidase/DAB, Rabbit/Mouse).

**Figure 4:**
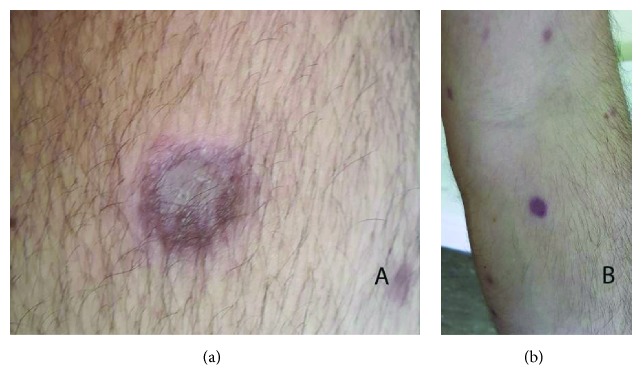
Complete remission of the eruption leaving depressed scars, including the previous tumor on the left thigh (a) and the papulonodular eruption at other body sites (b).

A 27-year-old male patient with a history of expanding skin lesions was referred to us for further management, after having received a 7-day per os antibiotic treatment with cefuroxime with no improvement. On physical examination there was a nodular, incipiently ulcerated, crumbly lesion 3 × 3 cm in dimension on the extensor surface of the left thigh, as well as crops of multiple elevated dome-shaped nodules and papules of reddish color and shiny, smooth surface on the trunk, the upper and lower extremities, the neck, periorbital and on the genitalia (Figures 1(a) and 1(b)). At some sites there was also a perilesional scaling. No pruritus was mentioned. The palms, the soles, and the mucous membranes were not involved. Moreover, a history of tiredness feeling in the previous months was mentioned. The history revealed though no consistent systemic B symptoms. The differential diagnosis included lymphomatoid papulosis, other cutaneous lymphomas, sarcoidosis, histiocytosis, and cutaneous infections (cutaneous leishmaniasis, atypical mycobacterial infection, and bacillary angiomatosis). Laboratory examination revealed an increased serum title of Bartonella quintana (1/80) and the patient received doxycycline 100 mg 1 × 2 for 2 weeks, with no change in a 2-week control examination. No other abnormal laboratory results were found (serology for CMV, EBV, HIV, HBV, HCV, Toxoplasma, Treponema pallidum, Leishmania, Coxiella burnetii, Mycoplasma pneumonia, Chlamydia pneumonia, Quantiferon test, RF, IgA, IgG, IgM, C3, C4, ANA, and ENA Screen). In the context of a possible histiocytosis we ordered an ophthalmological and an ENT consultation, both of which revealed no pathological signs. We performed two biopsies, one from the ulcerated lesion on the left thigh and another one from a dome-shaped but not ulcerated lesion on the left back. Bacterial, fungal, and mycobacterial cultures from the specimens were negative. Histology revealed a diffuse lymphocytic infiltration in the reticular dermis by small, medium, and large sized lymphocytes, some of which with anaplastic nuclei, accompanied by a variable number of neutrophils and histiocytes (Figures 2(a)–2(c)). Immunophenotypically the lymphocytes were CD3+ (Figure 3(a)), CD30+ (>75%, Figures 3(b) and 3(c)), CD4+, MUM1+, CD8-, ALK-1-, CD15-, and TIA-1-/+ (20% positive). The cellular marker for proliferation, Ki-67, was estimated ~ 80%. The epidermis was spared and showed a psoriasiform hyperplasia. Peripheral blood smear as well as immunophenotypic analysis of peripheral blood and bone marrow was performed with normal findings. The bone marrow biopsy showed absence of neoplastic infiltration, with minor deviations of the hemopoietic series of no particular significance. CT-scan of neck-thorax-abdomen showed no lymphadenopathy. The proposed diagnosis, based upon the correlation of clinical, histological, and immunophenotypical (expression of CD30) findings, was primary cutaneous CD30+ lymphoproliferative disorder with multifocal signs classified as lymphomatoid papulosis type A. According to the NCCN v2.2019 guidelines for primary cutaneous CD30+ T-cell lymphoproliferative disorders for adult patients with extensive disease, observation is preferred for asymptomatic patients [3]. Methotrexate (10 to 35 mg per week by oral or subcutaneous administration) is opted among other treatments in symptomatic patients and has been proposed to our patient due to the large ulcerative lesion on the left thigh and the extensive multifocal disease involving cosmetically important body areas. However, the patient refused to take any medication because of the potential adverse reactions of the therapy. At 4-week follow-up a striking improvement was observed, with the total of the lesions having regressed. Of notice was the regression of the ulcerated nodular lesion on the left thigh (Figures 4(a) and 4(b)). No new lesions were found. A complete response was observed at the follow-up visits, which remained in the last 6 month follow-up.

## 3. Discussion

Lymphomatoid papulosis is a rare, chronic, recurrent, self-healing disease with excellent prognosis in the majority of cases [4]. The list with the available treatment modalities includes apart from topical steroids [3], topical mechlorethamine [3], methotrexate [2, 5–7], targeted phototherapy [8], photodynamic therapy[9], oral or topical retinoids [10], and anti-CD30 monoclonal antibody-drug-conjugate (brentuximab vedotin) [11]. None of them changes actually the natural history of the disease or reduces the risk of developing an associated lymphoma [2]. However, they may hasten the healing of the lesions and prevent the eruption of new lesions [2]. In case of a limited or asymptomatic disease, a wait-and-see strategy is recommended. In terms of an extensive disease or a disease involving cosmetically sensitive areas (e.g. face)—as in our case—a low-dose methotrexate treatment is initiated, followed by a maintenance treatment [2]. Regarding our case of a 27-year-old male patient with extensive disease, involving cosmetically sensitive areas, a low-dose-methotrexate treatment was planned, in order to hasten the healing and preventing the eruption of new lesions, but was not accepted by the patient. The course of the untreated lesions, including the large ulcerated one, which usually may take months to heal, was impressively satisfying at the 4-week follow-up. The case should make us consider weighting the risks and the short-term benefits of methotrexate treatment, even in the case of an extensive disease. However, a situation like this should be systematically followed by the possibility of a relapse, the risk for second hematological neoplasia, and even development of a primary cutaneous anaplastic large cell lymphoma.

## References

[1] Kunishige J. H., McDonald H., Alvarez G., Johnson M., Prieto V., Duvic M. (2009). Lymphomatoid papulosis and associated lymphomas: a retrospective case series of 84 patients. *Clinical and Experimental Dermatology*.

[2] Kempf W., Pfaltz K., Vermeer M. H. (2011). EORTC, ISCL, and USCLC consensus recommendations for the treatment of primary cutaneous CD30-positive lymphoproliferative disorders: lymphomatoid papulosis and primary cutaneous anaplastic large-cell lymphoma. *Blood*.

[3] National Comprehensive Cancer Network Primary cutaneous CD30+ lymphoproliferative disorders. https://www.nccn.org/professionals/physician_gls/pdf/primary_cutaneous.pdf.

[4] Bekkenk M. W., Geelen F. A. M. J., van Voorst Vader P. C. (2000). Primary and secondary cutaneous CD30+ lymphoproliterative disorders: a report from the Dutch Cutaneous Lymphoma Group on the long-term follow-up data of 219 patients and guidelines for diagnosis and treatment. *Blood*.

[5] Vonderheid E. C., Sajjadian A., Kadin M. E. (1996). Methotrexate is effective therapy for lymphomatoid papulosis and other primary cutaneous CD30-positive lymphoproliferative disorders. *Journal of the American Academy of Dermatology*.

[6] Bruijn M., Horváth B., van Voorst Vader P., Willemze R., Vermeer M. (2015). Recommendations for treatment of lymphomatoid papulosis with methotrexate: a report from the Dutch Cutaneous Lymphoma Group. *British Journal of Dermatology*.

[7] Newland K. M., McCormack C. J., Twigger R. (2015). The efficacy of methotrexate for lymphomatoid papulosis. *Journal of the American Academy of Dermatology*.

[8] Kontos A. P., Kerr H. A., Malick F., Fivenson D. P., Lim H. W., Wong H. K. (2006). 308-nm Excimer laser for the treatment of lymphomatoid papulosis and stage IA mycosis fungoides. *Photodermatology, Photoimmunology & Photomedicine*.

[9] Rodrigues M., McCormack C., Yap L. (2009). Successful treatment of lymphomatoid papulosis with photodynamic therapy. *Australasian Journal of Dermatology*.

[10] Krathen R. A., Ward S., Duvic M. (2003). Bexarotene is a new treatment option for lymphomatoid papulosis. *Dermatology*.

[11] Duvic M., Tetzlaff M. T., Gangar P., Clos A. L., Sui D., Talpur R. (2015). Results of a phase II trial of brentuximab vedotin for cd30+ cutaneous t-cell lymphoma and lymphomatoid papulosis. *Journal of Clinical Oncology*.

